# Behavioral Medicine Physiotherapy in the Context of Return to Work for Chronic Pain: A Single-Case Experimental Design Study

**DOI:** 10.3390/ijerph19031509

**Published:** 2022-01-28

**Authors:** Hedvig Zetterberg, Ida Flink, Sören Spörndly-Nees, Sofia Wagner, Rolf Karlsten, Pernilla Åsenlöf

**Affiliations:** 1 Department of Women’s and Children’s Health, Uppsala University, 751 85 Uppsala, Sweden; soren.sporndly-nees@neuro.uu.se (S.S.-N.); sofia.wagner@neuro.uu.se (S.W.); pernilla.asenlof@neuro.uu.se (P.Å.); 2 The Center for Health and Medical Psychology, School of Law, Psychology and Social Work, Örebro University, 701 82 Örebro, Sweden; ida.flink@oru.se; 3 Department of Surgical Sciences, Uppsala University, 751 85 Uppsala, Sweden; rolf.karlsten@surgsci.uu.se

**Keywords:** behavioral medicine, chronic pain, return to work, rehabilitation, exercise

## Abstract

Effective interventions are needed for return-to-work (RTW) for individuals with chronic pain on long-term sick leave. In this study, a behavioral medicine physiotherapy protocol was systematically replicated and added to workplace components. The intervention was evaluated for fidelity and effects on target activities and work ability. A single-case experimental design was used with five participants. Daily and weekly ratings of personalized target activities at work as well as work ability were carried out throughout the study period of 26–28 weeks. Effects of the behavioral medicine physiotherapy intervention were evaluated for each individual using visual analysis of displayed graphs and quantitative non-overlap methods. Goal achievement for target activities was reviewed. Three participants completed the intervention. The results indicated an effect from the behavioral medicine physiotherapy intervention on task-specific self-efficacy for target activities, but no consistent effect on experience of target activities or work ability. All three participants had increased function in target activities in line with pre-defined goals. Fidelity to the intervention manual was good. Behavioral medicine physiotherapy can be successfully adapted to work disability and was here replicated in an RTW context for individuals with chronic pain. The intervention protocol should be further evaluated in large-scale studies.

## 1. Introduction

Chronic pain, i.e., pain lasting for more than 3 months [1], can contribute to substantial disability and suffering for the individual and is associated with poor health outcomes [2] and decreased work ability [3,4]. Chronic pain is common in adult populations, with a prevalence of 19% [3]. Pain-related disorders constitute the second most common cause of long-term sick leave in western countries [5]. In Sweden, specifically, 17% of long-term sick leave is related to musculoskeletal diagnoses [6]. Effective interventions for return-to-work (RTW) are needed for individuals with chronic pain on sick leave. Although there are numerous intervention programs in different contexts for individuals on sick leave, there seem to be diverse outcomes regarding RTW [7], with consistent support only for interventions with active involvement in the workplace [8,9,10]. Integration of actions to support RTW for individuals with chronic pain within primary care is a promising way forward, where more research is needed.

Key components in RTW processes include an active dialogue between the employee on sick leave and the immediate supervisor at work, and a joint problem-solving and action plan [11]. Integrating psychosocial and behavioral interventions has been suggested for more comprehensive RTW interventions [10,11,12]. Some examples are multi-domain interventions (e.g., healthcare and work accommodations), multidisciplinary treatment and physiotherapy combined with workplace dialogue, which have demonstrated an effect on RTW in different pain conditions [10,13,14,15]. There are also effective approaches of combining workplace and supervisor involvement with graded activity or psychological and physiotherapy treatment [16,17]. Social context, organization, and stakeholders related to sick leave and RTW interventions differ between countries, meaning increased efforts in RTW interventions could look different. In Sweden, implementation of RTW coordinators in primary care is ongoing; however, the content of interventions is not standardized and ranges from administrative support to individualized case-management.

In behavioral medicine physiotherapy, treatments are based on biopsychosocial assessment, including cognition, behavioral contingencies, and context [18,19]. The integration of behavioral and biomedical knowledge forms the theoretical basis [20]. The treatment approach in the protocol by Åsenlöf et al. [18] aims to decrease patients’ disabilities by tailoring assessment and treatment to individually prioritized activities in everyday life, i.e., target activities where the patient perceive hinders and wants to increase activity performance. Behavior change strategies are combined with physical exercise, and tailored behavioral skills training is a key component. To provide behavioral medicine physiotherapy, physiotherapists receive training in the approach, including the biopsychosocial model, behavioral analysis and learning theories. To facilitate health-related behavior change, active strategies such as goal-setting, feedback, shaping, graded tasks and social support are applied [21]. Training is often provided as a university course or as part of an implementation program. This modern approach within physiotherapy has also been implemented in the undergraduate physiotherapy program curriculum in two universities in Sweden [22]. The behavioral medicine physiotherapy has similarities to other combinations of physical exercise and psychosocial interventions for chronic pain and have demonstrated effects on disability in various pain conditions [18,19,23,24,25].

Psychosocial factors are known to play a major role in pain-related disability [26,27], with self-efficacy and fear-related avoidance of activities as important components [28,29]. Treatment programs for pain conditions that incorporate psychosocial factors result in decreased disability compared to ordinary primary care or physical exercise only [15,18,30,31,32,33]. The biopsychosocial approach to chronic pain is implemented in both specialized and primary care [15,33], with implementation in physiotherapy practice [19,33]. Regarding physical exercise, individuals with chronic pain are recommended regular physical activity to decrease the risk of ill-health [2,34,35], and exercise programs might be beneficial for the pain condition [36].

Behavioral medicine physiotherapy in the protocol developed by Åsenlöf et al. [18], has been demonstrated to effectively improve disability and goal achievement among individuals with musculoskeletal pain [18,23,37,38]. This protocol has not been previously implemented with a focus on work ability for individuals with chronic pain on sick leave and tailored to target activities at work. Based on the need for work-directed rehabilitation programs, a relevant research question arises as to how components of workplace involvement, and behavioral medicine physiotherapy could be combined for individuals with chronic pain on long-term sick leave. Here, behavioral medicine physiotherapy, previously evaluated by Åsenlöf et al. [18,23], is systematically replicated in the context of RTW interventions involving the workplace, and developed to needs in work-directed rehabilitation.

In this study, a combined intervention for individuals with chronic pain is provided, where workplace components form the basis, including RTW coordination with a workplace meeting [39], and a novel approach of skills training in communication and problem solving between employee and supervisor [40]. The added behavioral medicine physiotherapy intervention is tailored to target activities at work with the aim to improve activity performance. The rationale for the combined protocol [18,39,40] is the importance of workplace involvement, i.e., this was considered to be the context when applying work-directed behavioral medicine physiotherapy for individuals on long-term sick leave. That is, workplace dialogue was considered necessary for an RTW plan and for eventual work adjustments. The behavioral medicine physiotherapy intervention was hypothesized to improve pain-related disability, here in activities related to work tasks, which were considered a necessary component for RTW for individuals with chronic pain. Specifically, it was hypothesized that the physiotherapy intervention would improve individual’s target activities, and that this might also affect work ability. A single-case experimental design was applied to gain detailed insight into target activities at work and work ability over time in each individual.

Specific research questions were:

- Is there an effect of the behavioral medicine physiotherapy intervention, when added to workplace components, on personalized target activities and work ability for each individual?
- What is the goal achievement for the personalized target activities at work, for each individual?
- What is the fidelity of the combined intervention protocol?

## 2. Materials and Methods

This study was part of a larger trial about the effect of behavioral medicine physiotherapy on RTW in patients with chronic pain. The project was registered at ClinicalTrials.gov (NCT03827174) (accessed on 4 October 2021) and approved by the Regional Ethical Review Board in Uppsala, Sweden, (Number 2018/435). In this study, the intervention protocol was developed and evaluated for five individuals, applying a single-case experimental design. Reporting of this study has been guided by the Single-Case Reporting Guidelines In BEhavioral Interventions 2016 (SCRIBE) [41]. All participants received oral and written information and gave written informed consent prior to study participation. The recruitment and the intervention period for this study were from August 2019 to June 2020. Due to the outbreak of the COVID-19 pandemic, the intervention was delivered online from March 2020.

### 2.1. Study Design

An ABCD + follow-up design was used, replicated across five individuals with varying lengths of baselines and intervention phases. The design was non-concurrent, with participants starting their baselines at different time-points. Single-case experimental designs (SCED) build on repeated measures for the individual throughout a study period with defined phases, which allow for experimental evaluation at the individual level [42]. The design allows an intervention to be tailored to the individual, and it is suitable for evaluation of new interventions and provides insight into process variables over time [43,44]. Functional relationships between intervention and outcomes are evaluated visually, based on the prospective data over phases, and can be combined with quantitative effect measures [45]. In this study, the behavioral medicine physiotherapy intervention, phase D, was evaluated as added to preceding phases A–B–C: the initial baseline (A) and the workplace components; return-to-work coordination (B), and effective communication within the organization (C). Follow-up periods were post intervention (PI) and at 1 and 2 months (F1, F2). Baseline lengths were initially randomized; however, a complete randomization of study lengths could not be implemented due to characteristics of the intervention and the clinical setting. The phases had the following lengths and varied across participants: (A) 2–3 weeks, (B) 2–5 weeks, (C) 3–5 weeks, and (D) 14–18 weeks. The combined A–B–C length varied between 9 and 12 weeks and the behavioral medicine physiotherapy intervention (D) was analyzed in relation to the combined preceding phases.

### 2.2. Participants and Setting

When using SCED, it is common to strive to include typical clinical cases [42]. Hence, the inclusion criteria were >18 years old, pain for more than 3 months, and long-term (>30 days) full-time or part-time sick leave from salaried employment or studies. In the recruitment process, it was also stressed that this study targeted individuals who had a wish to increase their hours at work with at least 25%. Exclusion criteria were severe psychiatric illness, severe substance use disorder, and orthopedic surgery in the past 3 months. In addition, participants whose immediate work supervisor did not agree to take part in the interventions were excluded. Five participants were included. They were recruited from a pain center at a university hospital and from a primary care unit in Sweden. Information about the study was posted at the clinical centers, where the personnel informed eligible patients. Individuals who expressed interest were contacted by the study researchers and received detailed information about the study. After initial consent from the participant, his or her immediate supervisor at work was contacted and asked to participate.

### 2.3. Measures

#### 2.3.1. Procedures

At the first research visit, the participant was asked to prioritize personalized target activities at work affected by pain-related disability, which they wanted to improve. Participants were asked, “Which activities are important for you in your work?”. These activities were rated according to a modified version of the Patient Goal Priority Questionnaire (PGPQ), restricted to work activities only [38]. Target activities in daily life for a person with chronic pain could be driving the car or playing with the kids in a park. Target activities at work should be related to work tasks and could be to lift packages or to sit by the computer. Repeated ratings of the personalized target activities and work ability were carried out daily and weekly throughout the study period via online administered questionnaires, sent to the participants by e-mail or text messages. Descriptive pre- and post-measures were administered at baseline and post-intervention, and at 2-month follow-up. An overview of design phases and reported measures can be found in Table 1.

#### 2.3.2. Repeated Ratings of Target Activities and Work Ability

Self-efficacy for target activities was rated weekly, with the item, “How confident are you with your ability to (target activity)?” rated on a numeric rating scale (NRS) 0–10, ranging from “very unconfident” to “very confident.”

Experience of daily target activities was rated with the item, “How did (target activity) go today?” on a NRS 0–10, ranging from “not at all” to “excellent.” This information about the rating was collected on each workday, on days when the target activity had been performed.

Work ability was rated on each workday with an item from the Work Ability Index [46], modified to today, “Assume that your work ability, at lifetime best, is valued at 10. What score would you give your work ability today?” rated on an NRS 0–10, ranging from “completely unable to work” to “my work ability is at its best”.

#### 2.3.3. Goal Achievement for Target Activities and Self-Rated Improvement

Reported frequency or duration, in numbers or minutes, of the target activities was registered on each workday. Data were used for evaluation of goal achievement in relation to pre-defined goals, for example, to increase the amount of an activity at work.

Additional measures of goal achievement and clinical improvement were the modified Patient Goal Priority Questionnaire (PGPQ) and the Patient Global Impression of Change (PGIC) which were administered at baseline, post-intervention, and at 2-month follow-up. The PGPQ is a measure developed for behavioral goal assessment and for use as a tailored outcome measure. Items on activity performance (ranging from no disability to not able at all) and satisfaction with ability for the target activities were used for goal achievement in this study. Each item is rated on NRS 0–10. The PGPQ has been evaluated with acceptable validity and reliability [38,47]. The Patient Global Impression of Change is a measure designed to assess patients’ perception of changes in health status [48]. Patients rate their change as “very much improved”, “much improved”, “minimally improved”, “no change”, “minimally worse”, “much worse”, or “very much worse”. The two highest ratings have been demonstrated to indicate important and substantial improvement [48].

#### 2.3.4. Intervention Fidelity

The following aspects of fidelity [49] were evaluated for the combined intervention protocol:

Study design: adherence among participants and providers to study design and intervention protocol was monitored continuously from study logs and follow-up meetings with treating personnel. Intervention checklists from the providers, and participants’ personalized treatment plans were also evaluated for consistency with intervention components: templates from the workplace meetings, exercise programs, specified goals for target activities, and individualized home assignments.

Delivery of intervention: study logs and intervention checklists were used to evaluate whether the content and dose of intervention were delivered as specified. Study logs, participants’ exercise programs and diary for home assignments in the physiotherapy intervention provided information regarding adherence to the intervention components by participants.

Receipt and enactment of intervention: An evaluation form completed by the participants at the end of the physiotherapy intervention regarding the skills learned, own reflections, and plan for maintenance provided information about the participants’ understanding of the intervention.

Each participant could report on adverse events in a free text question in daily and weekly questionnaires, or directly to the treating personnel. In addition, all personnel had training in the theoretical underpinnings of the intervention and its protocol. Treatment manuals were used for all intervention components.

#### 2.3.5. Descriptive Pre- and Post-Measures

Information related to sick leave, work, and self-efficacy to increase time at work was collected using self-reported items in the online questionnaires.

Pain intensity was assessed with an item modified from the Brief Pain Inventory [50], “Please rate your pain by choosing the number that best describes your pain intensity at its worst in the last 24 h”, rated on NRS 0–10, ranging from “no pain”, to “pain as bad as you can imagine”.

The Work Ability Index (WAI) was used to measure work ability, in addition to daily ratings. The WAI measures work ability in relation to demands at the workplace, as well as the worker’s health state and mental resources [46]. A total index is computed ranging from 7 to 49, where higher scores indicate better work ability. The WAI has acceptable validity and reliability [51,52].

Pain Disability Index (PDI) measures pain-interference in the following areas: family and home responsibilities, recreation, social activity, occupation and education, sexual behavior, self-care, and life-support activity. Each item is rated on NRS 0–10, ranging from “no disability” to “worst disability”. Total score ranges from 0 to 70, with higher scores reflecting higher interference of pain with daily activities. The PDI is a valid and reliable measure [53,54].

### 2.4. Intervention

#### 2.4.1. Return-to-Work Coordination (RTW-C)—Phase B

The first workplace component aimed to facilitate coordination between stakeholders regarding RTW, and a dialogue between participant and immediate supervisor at work. The initial steps were screening with the participant, and coordination with the physician and the Social Insurance Agency, according to needs. In a workplace meeting between participant and his/her supervisor, a common RTW plan was discussed, including work modifications and support in the workplace. Details about the specific action plan and time for follow-up were decided upon between the participant and the supervisor and documented according to a template from the Swedish Social Insurance Agency. The coordinators were clinical healthcare personnel, e.g., physiotherapists, with standardized training from the County Council in legislations regarding sick leave and the needs and actions of RTW coordination [39].

#### 2.4.2. Effective Communication within the Organization (ECO)—Phase C

The second workplace component aimed to target psychosocial factors at the workplace, and was based on a protocol from a previous study: the Effective Communication within the Organization (ECO), originally developed as a selective prevention for employees with pain related ill-health [40]. The ECO is a brief psychosocial intervention where supervisors and their employees are trained in supportive communication skills and problem solving, which aims to target psychosocial factors and to facilitate supervisor-employee interaction.

In this study, a modified version of the ECO-protocol was developed to build on the RTW-C. Participants and their supervisors took part in two sessions separately: (1) Communication, including validation with homework of skills-training (supervisors) and self-validation (participants) and (2) Problem solving, focused on factors that can be influenced at the workplace. Thereafter, a second workplace meeting was conducted to follow-up on and revise the return-to-work plan from the RTW-C meeting and plan for a continued dialogue at the workplace. The program was provided by a physiotherapist and a clinical social worker, who had been provided training in the manual by members of the research group who developed the first version of the protocol [40].

#### 2.4.3. Behavioral Medicine Physiotherapy—Phase D

The behavioral medicine physiotherapy intervention was built on a previously studied protocol by Åsenlöf et al. [18,37], which was developed and replicated here in an RTW context. The aim of the behavioral medicine physiotherapy was to decrease pain-related disability and improve target activity performance. A structured description of the intervention protocol according to the Template for Intervention Description and Replication Checklist can be found in Supplementary materials.

A core feature of the behavioral medicine physiotherapy intervention was the identification of individually prioritized target activities, to which biopsychosocial assessment and treatment is tailored. After analysis and goal setting, a physical exercise and behavioral skills training period follows, see detailed description below. Since the previous studies, the protocol was developed as follows: As an adaptation to the RTW context, target activities in daily living were here limited to target activities at work. That is, in this study, individuals were enquired to prioritize activities at work, affected by pain disability, which they wished to improve. To ensure an evidence-based level of physical exercise [2,34,35], the amount and intensity of aerobic and strength training were monitored and progressively increased. This was performed for the general aerobic and strength training program, motivated by the beneficial effects of physical activity in chronic pain conditions [36,55], but also for the physical exercises motivated by prioritized target activities at work and associated functional behavioral analyses.

The behavioral medicine physiotherapy was structured into a 14-week program. Initially, a brief assessment was made regarding the participant’s beliefs in pain and an education in pain neurophysiology, the biopsychosocial model of chronic pain, rationale for physical exercises and approach to pain was provided [55,56]. The exercise program was delivered in group sessions at a training facility with supervision from a physiotherapist, combined with individual follow-up on individualized exercises and home assignments every second week.

Tailored exercises and behavioral skills training. The following structure, which is similar to the original protocol by Åsenlöf et al. [18,37], formed the basis for the treatment: (1) identification of personalized target activities at work and perceived problems; (2) assessment and self-monitoring; (3) functional behavioral analysis; (4) goal-setting and treatment plan; (5–7) basic, applied, and generalized skills training; (8) maintenance and relapse prevention; (9) evaluation. Participants monitored the target activities for behavioral contingencies, including thoughts and emotions. A functional behavioral analysis was conducted by the physiotherapist based on interview with participant and results of the monitoring. Analysis of physical function in relation to target activity was included when applicable. Goal-setting of target activities was specified and agreed upon between the participant and the physiotherapist, and new goals could be added [57,58]. Based on the functional behavioral analysis, physical function, and task-analysis of the target activity, exercises for basic and applied skills training were chosen by the physiotherapist and the participant together. Basic skills training could include acquisition of skills required in the target activity, practiced separately outside of the work context. Physical aspects could be function required in activity or improved physical prerequisites, and psychological aspects could be identification of thoughts and actions and exposure for pain-related fear. Examples included neck and shoulder exercises, increased load in exercises, exposure, and coping strategies such as taking breaks or practice of self-regulation, etc. Applied skills training included real-life practice of basic skills in target activities at work, and generalization over contexts. That is, application of physical or cognitive strategies during target activities, and a graded increase in the target activity in terms of duration or frequency. Examples included exposure in applied context at work, application of strategies in target activities at work, and sub-goals for graded increase of activity. Behavioral treatment strategies, such as exposure to feared activities, were incorporated in the basic and applied skills training, if functional analysis indicated fear-related avoidance [26,57]. Finally, at the end of the physiotherapy intervention, the participants were encouraged to plan for maintenance of both regular physical activity and target activity skills at work, including a plan for relapse prevention [59]. Description of the functional behavioral analyses and treatment content for the participants can be found in the Results section.

General aerobic and strength training: Exercise sessions were scheduled twice a week, with each session lasting 75 min. The aerobic exercise part comprised 20 min at moderate intensity (70% of maximum heart rate). In addition, participants were recommended to engage in moderate aerobic activity between sessions, to reach a total of 150 min/week. Participants received a Huawei Honor Band 5, an accelerometer, to encourage self-monitoring of their aerobic activity. The exercise program was constructed to be in line with recommendations of 150 min of moderate intensity or 75 min of vigorous intensity per week and strength training two days a week [34,35]. The strength training consisted of a whole-body approach, including leg, upper body pull and push, shoulder and core exercises. Exercises were adjusted to the individual based on physical function and preferences. Pain during exercises was allowed, and exercises were agreed by participants. Screening for fear of pain related to the physical exercise components was performed initially by a questionnaire made for this study, where participants rated fear for each exercise on a NRS 0–10. When fear was reported, a similar approach as described above was implemented, with self-monitoring, exposure and graded increase in the exercise. The participants were instructed on adjusting resistance in order to perform repetition maximum (RM) in each set and increase resistance when capable to perform more than the prescribed repetitions. Stepped increase in intensity was as follows: 15 RM weeks 1–4, 8–12 RM weeks 5–8, and 6–8 RM weeks 9–14. As an additional indication of intensity of resistance exercises, the participants were instructed that the training should be at an intensity perceived as challenging, corresponding to 7 or above at the Omni-scale of Resistance Exercise, NRS 0–10 [60]. Participants were instructed to engage in aerobic training at an intensity corresponding to 11 or above on the Borgs RPE scale [61].

All physiotherapists had several years of clinical experience and special training in behavioral medicine physiotherapy. Training included a post-graduate university course (credits corresponding to five weeks of full-time studies) in behavioral medicine and its applications in physiotherapy, including functional behavior analysis and behavior change techniques. The course is provided at Uppsala University, Sweden. In addition, introduction to the manual in this study was provided and meetings with the treating physiotherapists were held regularly. Supervision was provided within the research team by senior personnel, and consistency to the previously studied protocol [18,37] was ensured by the last author.

### 2.5. Data Management and Analyses

For data collected daily, the median scores were calculated for each week and used in further analyses. Ratings of target activities and work ability were analyzed for participants who completed the full study protocol.

The response rate for each participant was calculated as percentage answered questionnaires. Participants were instructed to submit their daily questionnaires within a timeframe of 24 h. The percentages were calculated for the responses fulfilling the criteria. Since data were collected only during workdays, according to a daily timeline, there could be additional missing values for repeated ratings due to planned vacation or short-term sick leave. For target activities, answers were also dependent on if the target activity had been performed, resulting in an uneven frequency of the data sample, partly due to the participant’s work tasks.

Visual analysis formed the basis for evaluation of the repeated ratings of target activities and work ability. Data were displayed in separate graphs with inserted lines for level and trend for each phase, to facilitate the visual analysis [43]. The behavioral medicine physiotherapy intervention, phase D, was analyzed in relation to all preceding phases (A–B–C). The maintenance of potential effects during the follow-up period was also analyzed. In addition, components within the physiotherapy phase were analyzed according to the time of their presentation. Focusing on parts of the observations can be justified when latency of change or gradual improvement can be expected [42]. A structured visual analysis was conducted according to steps as described by Kratochwill et al. [45]. First, patterns of data (stability, predictability) were evaluated. Second, within- and between-phase data patterns were examined according to: (a) level, (b) trend, (c) variability, (d) immediacy of the effect, (e) overlap, and (f) consistency of data patterns across similar phases. Finally, information from all participants were integrated to determine if there were at least three demonstrations of effect. Two of the researchers (HZ, SW) performed the analyses independently and then discussed their individual findings until a consensus was reached. Visual analyses were also cross-checked by the other co-authors.

As a complement to visual analyses, quantitative effect measures were calculated for target activities and work ability. Nonoverlap methods were chosen due to assumptions of delayed effects and large variability in data, in addition to the data being rated on ordinal scales. The Nonoverlap of All Pairs (NAP) is a method for measuring the percentage nonoverlap for two phases [62]. To address the potential positive trend at baseline, the Tau-U measure was also used. Tau-U builds on Kendall’s rank correlation and is a method that evaluates improvement of data points between phases, similar to the NAP [63]. In addition to nonoverlap, Tau-U calculates trends in data, and a control for the trend at the baseline phase can be added. The Tau-U values range from −1 to 1, interpreted as percentage of data showing improvement between phases. An online calculator was used to calculate the NAP and Tau-U scores [64]. Correction for baseline trend was used when the baseline trend Tau was 0.2 or above [65]. The following ranges were used for NAP and Tau-U values as effect sizes: weak 0 to 0.65, medium 0.66 to 0.93 and large 0.93 to 1.0 [62].

The goal achievement for target activities was reported by the behavioral outcome of frequency or duration of target activities in relation to goals. Repeated measures of frequency or duration of target activities were extracted for a two-week period at each time-point, and median values calculated.

For descriptive pre- and post-measures, total scores on questionnaires were calculated when applicable. Furthermore, for data collected throughout the study period, mean or median from a 2-week time-period during baseline, post-intervention, and 2-month follow-up were calculated in Excel 2016 (Microsoft, Redmond, WA, USA).

## 3. Results

Out of the five participants, two (P2 and P4) dropped out before or at the beginning of the behavioral medicine physiotherapy intervention. They reported lack of motivation or time to engage in the physical exercise program. The other three participants (P1, P3, P5) completed the full study protocol. P1 had a response rate of 74.2% for daily and 90.3% for weekly surveys. P3 had a response rate of 98.1% for daily and 100% weekly surveys, and P5 had a response rate of 100% for daily and weekly surveys. For P1, 87.0% of the daily surveys were submitted within a time frame of one day; moreover, for P3 and P5, the numbers were 97.6% and 100%, respectively.

### 3.1. Participant Characteristics and Descriptive Pre- and Post-Measures

Among the five participants, there were four women and one man. Mean age was 43 years, ranging from 24–63 years. All were employed or studying, and on part-time sick leave. An overview of participant characteristics can be found in Table 2. In Table 3, descriptive measures on work time and sick leave, confidence in increasing time at work by 25%, and total score on the WAI are presented for baseline, post-intervention, and 2-month follow-up. In Table 4, pain-related disability and pain intensity are presented for the corresponding time-points, as well as physical activity.

### 3.2. Repeated Ratings of Target Activities and Work Ability, Goal Achievement for Target Activities and Self-Rated Improvement

**Figure 1:**
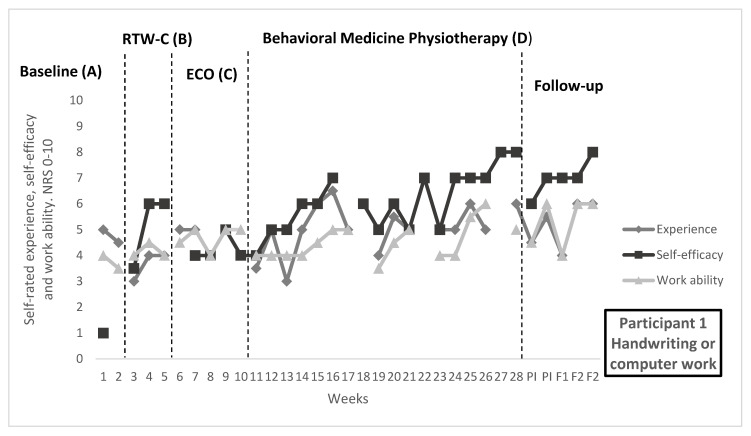
Target activity and work ability ratings for participant 1. Experience of and self-efficacy for the target activity “Handwriting or computer work”, as well as general work ability, rated on numerical rating scales 0–10, where higher score indicates better outcome. Median score per week are displayed for full study period, including baseline (**A**), return-to-work coordination (**B**), effective communication within the organization (**C**), behavioral medicine physiotherapy (**D**), post-intervention, 1-month follow-up and 2-month follow-up. During the physiotherapy intervention, on-set of applies skills training was week 18, generalization week 20, and maintenance and relapse prevention week 26.

**Figure 2:**
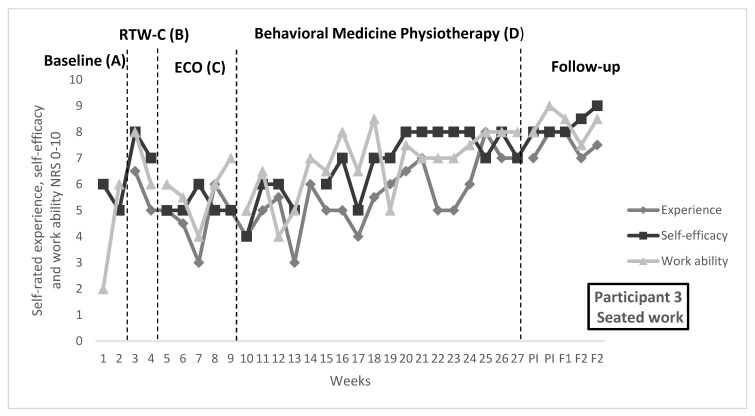

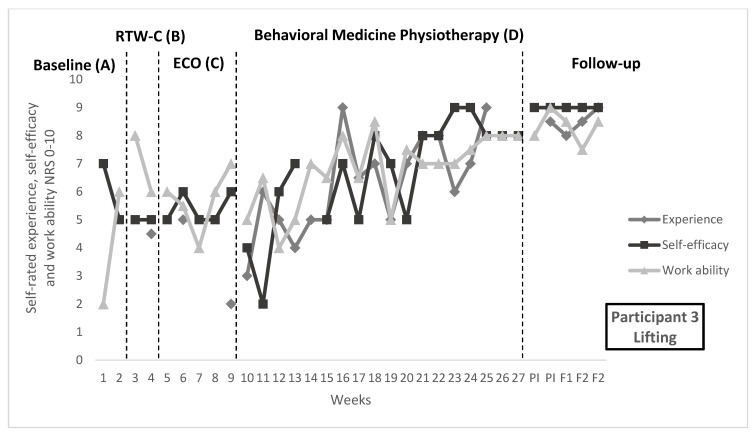
Target activity and work ability ratings for participant 3. Experience of and self-efficacy for the target activities “Seated work” and “Lifting”, as well as general work ability, rated on numerical rating scales 0–10, where higher score indicates better outcome. Median score per week are displayed for full study period, including baseline (**A**), return-to-work coordination (**B**), effective communication within the organization (**C**), behavioral medicine physiotherapy (**D**), post-intervention, 1-month follow-up and 2-month follow-up. During the physiotherapy intervention, on-set of applies skills training was week 17, generalization week 19 and maintenance and relapse prevention week 25. Change of sick leave and work context were at the following time points: week 10, week 26 and F1.

**Figure 3:**
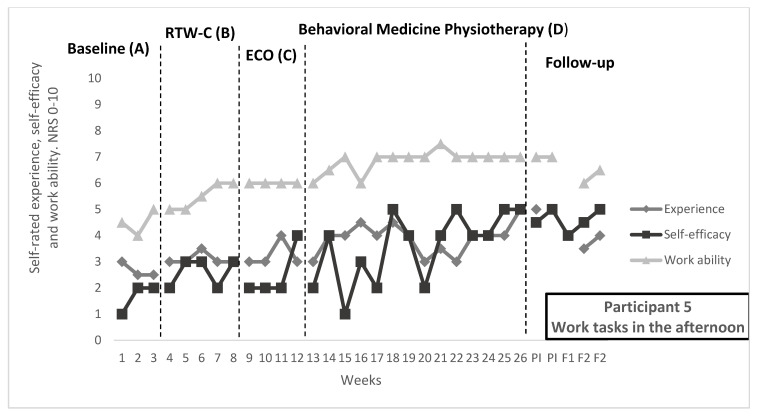
Target activity and work ability ratings for participant 5. Experience of and self-efficacy for the target activity “Work task in the afternoon”, as well as general work ability, rated on numerical rating scales 0–10, where higher score indicates better outcome. Median score per week are displayed for full study period, including baseline (**A**), return-to-work coordination (**B**), effective communication within the organization (**C**), behavioral medicine physiotherapy (**D**), post-intervention, 1-month follow-up and 2-month follow-up. During the physiotherapy intervention, on-set of applied skills training was week 20, generalization week 23 and maintenance and relapse prevention week 25.

In Figure 1, Figure 2 and Figure 3, repeated ratings of target activities and work ability are visualized in individual graphs for participants 1, 3, and 5. Median and range per measure and phase, as well as NAP and Tau-U values, are reported in Table 5. Goal achievement for each individual can be found in Table 6, as well as the self-rated improvement. Table 6 also contains description of functional analyses and treatment content in the behavioral medicine physiotherapy intervention, related to the target activities.

#### 3.2.1. Participant 1

Work demands for participant 1 included attending lectures and writing.

Effect on repeated ratings: An improvement in the self-efficacy for the target activity “Handwriting or computer work” was observed during the behavioral medicine physiotherapy intervention, in the visual analysis. Variability was seen in previous phases with higher ratings during the RTW-coordination. During the physiotherapy intervention, a positive trend and an increased level were seen, which were maintained during follow-up. No effects were seen for the experience of target activity, or for work ability according to visual analysis.

Goal achievement: For participant 1, the goal of being able to do the activity “Handwriting or computer work” for 45 min (a lecture) was achieved, and an increased duration of the activity was reported. Participant 1 rated improved performance of the target activity after the intervention period as well as increased satisfaction with ability for the target activity. During the time period, participant 1 also increased study time per day and credits taken, see Table 3.

#### 3.2.2. Participant 3

In the participant’s work as a health care professional, work demands included both seated work and more physically demanding tasks such as lifting.

Effect on repeated ratings: Improvement of self-efficacy for both target activities, “Seated work” and “Lifting”, was observed during the behavioral medicine physiotherapy intervention, in the visual analysis. During the physiotherapy intervention, a large variability in self-efficacy rating was seen initially for both activities. From about the middle of the physiotherapy intervention, less variability and improvement in terms of trend and level were observed. Higher levels of ratings at the end of the physiotherapy intervention were maintained, or further increased, during follow-up. In visual analysis of experience of target activities, both activities seemed to have improved during the physiotherapy intervention in terms of increased level and trend. However, the analyses are more uncertain due to unstable patterns and high variability. For ratings of experience, follow-up phase showed less variability. Effect on work ability was uncertain according to the visual analysis, with large variability and overlap in data, although a stabilization at a higher level was seen at the end of the physiotherapy intervention and follow-up.

Goal achievement: For participant 3, the goal was to perform target activities based on the needs at work. After the intervention period, the duration and frequency (respectively) of each target activity had increased, in line with the pre-defined goals. A change in the work context should also be noted. During the time period, participant 3 increased work time (see Table 3) and went from adjusted work tasks back to ordinary work tasks with more physical demands. Participant 3 rated improved activity performance and satisfaction with ability for target activities after the intervention period.

#### 3.2.3. Participant 5

For participant 5, who worked with administration, the pain intensity increased during the day; in the afternoon, the work tasks were perceived as difficult.

Effect on repeated ratings: Improvement of self-efficacy for the target activity “Work tasks in the afternoon” was observed during the behavioral medicine physiotherapy intervention, in the visual analysis. A large variability in the beginning of the physiotherapy intervention, and a positive trend just before the start of the phase, could be noted. Self-efficacy ratings were stabilized at a higher level at the end of the physiotherapy intervention and maintained during the follow-up. For experience of the target activity, there was uncertainty in the visual analysis due to high degree of overlap and low increase in the level and trend. Work ability had a positive trend and increased levels during the full study period, which is why specific effects of the physiotherapy intervention could not be noted. However, downward trends were noted during the follow-up for work ability ratings and experience of target activity.

Goal achievement: For participant 5, the goal was to be able to do more cognitively demanding work tasks in the afternoon, since work ability was perceived as worsening during the day. The frequency in doing these more demanding tasks had increased after the intervention period. Participant 5 rated improved activity performance and satisfaction with ability for the target activity after the intervention period, however with a remaining moderate degree of limitations in activity.

### 3.3. Intervention Fidelity

Study design: The combined intervention was consistent with the design and the protocol. Decisions were made to develop the protocol for the behavioral medicine physiotherapy intervention during the study period, based on intermediate analyses of target activities and goal-setting which called for further development. Changes in the protocol included: extended physiotherapy intervention period for participants 1 and 3, more structured sub-goals of graded increase in target activities, modified home assignments for target activities, and weekly feedback to participants with their own data on target activity frequency/duration and experience. In addition, delivery of an online intervention was decided upon for participant 5 due to the COVID-19 pandemic.

Delivery of intervention: All five participants took part in the full program in RTW-C and ECO. For the three participants who completed the physiotherapy intervention, adherence to the exercise program by the participants can be found in Table 7. Home assignments were completed by all three participants in the physiotherapy intervention.

Receipt and enactment of intervention: All three participants who completed the protocol engaged in the target activities at work and applied home assignments in their daily work context. Written reports from participants 1, 3, and 5 showed the following examples of skills learned during interventions:

P1: “Taking regular breaks, keeping my concentration during the lectures, being able to sit for a longer period of time.” “My body can handle more than I think. Not to be afraid of doing something ‘wrong’.”

P3: “To be able to sit for a longer period of time, to do heavier lifts, extended work tasks, and have longer workdays.” “If I do something I think/know is bad for my pain, is it ok to do it anyway. I don’t have to adapt as much as I have done earlier; it did not make my daily life any easier, rather harder!”

P5: “Taking regular breaks. Challenging myself with harder work tasks in the afternoon.” “Small improvement are also improvements.”

## 4. Discussion

As a summary, an effect from the behavioral medicine physiotherapy intervention was noted on task-specific self-efficacy for target activities for each of the three individuals who completed the study protocol. For the experience of daily target activities, an improvement was seen in one of the three participants (P3). Work ability seemed to improve during the study period for two participants (P3, P5); however, it is not certain that these changes were associated with the physiotherapy intervention. Goal achievement was reported, in line with the pre-defined goals for the respective activity. The results indicate that, although frequency or duration increased and self-efficacy for target activities improved, the experience of the target activities did not consistently follow the same pattern and might thus be affected by other aspects. The daily experience of performing activities at work, when suffering from chronic pain, need to be further elaborated. Presumably, the experience could be affected by the lived experience of pain, associated thoughts and emotions, and the context [66], and differentiated from the self-efficacy for performing the activity.

Results of the self-efficacy ratings were supported by measures of global improvement and target activity performance and satisfaction, where a pattern of improvement was seen across items for all three participants. Generalization measures can be seen as validating, adding to the individualized repeated measures in SCED [43]. Here, the rated improvements of target activity performance could correspond to a reliable change [47], and the global rating has previously been reported to correspond to improvements in daily life activities [67]. The complementary quantitative nonoverlap methods (NAP and Tau-U), indicating improvement between phases, showed moderate to large effect sizes for follow-up compared to the A–B–C phases, for all outcomes which were considered as improvements according to the visual analyses.

In this study, we replicated a previously evaluated behavioral medicine physiotherapy protocol in an RTW context for individuals with chronic pain and adapted it to work disability needs. The results indicate a promising effect of the behavioral medicine physiotherapy intervention on personalized target activities at work. These findings are in line with previous studies of the behavioral medicine physiotherapy by Åsenlöf et al., which displayed improved performance in target activities and decreased disability [18,37,38]. Hence, development of the manual to an RTW context can be considered promising. Fidelity to the intervention manual was good, further supporting the developed protocol. The behavioral medicine physiotherapy was here combined with components of active workplace involvement, the RTW coordination and the ECO, which both contained a meeting and action planning at work between participant and immediate supervisor. Interaction within the intervention parts is not known, and effects could be combined. However, the self-efficacy ratings changed during the middle of the physiotherapy intervention, supporting the interpretation that the behavioral medicine physiotherapy intervention affected task-specific self-efficacy. For work ability though, a pattern of improvement was seen for some participants during the workplace phases or the full study period, which is why a potential effect could not be related to the physiotherapy intervention separately. The potential of combined effect on work ability need to be further studied. In addition, information on adherence to the action plans made during the meetings between participant and supervisor are lacking, which would be of interest for process analyses.

The behavioral medicine physiotherapy intervention was hypothesized to affect personalized target activities by a combination of structured exercises and tailored behavioral skills training, with the aim of long-term maintenance. A core feature of the treatment approach is applied skills training, i.e., basic skills acquired (physical or cognitive) are practiced in the target activities in everyday life setting, in this study at the workplace. Applied skills training also includes a graded increase in the target activity. Previous research on the behavioral medicine physiotherapy protocol have shown that change of target activities occurred during the applied skills training phase [37], as replicated in this study. This indicates that guidance on implementation of skills in activities in everyday life is valuable in rehabilitation, to decrease disability for individuals with chronic pain. Applied skills training could include, but is not limited to, specified sub-goals for amount of target activity, which has similarities to other protocols with graded activity. Previous research has demonstrated effect of graded activity programs on pain disability [68], including application to work context [16]. In the applied skills training, the approach of graded activity could be combined with exposure for fear of pain.

Task-specific self-efficacy was related to behavioral performance and function of target activities. Self-efficacy can be interpreted as a potential mediator of intervention effect, which could be supported by the consistency between goal achievement and self-efficacy as indicated by the ratings. There was also a pattern of improvement of self-efficacy during the applied skills training, which could be interpreted as increased self-efficacy might relate to acquired skills necessary for goal achievement. According to Bandura, “mastery experience” is a source of self-efficacy, which is coherent to the results in this study [69]. A large body of research has evaluated the predictive value of self-efficacy for pain-related outcomes [28,70,71], and self-efficacy has been suggested to mediate the relationship between pain and activity performance and function [72,73].

In this study, we tested the preliminary effects of a comprehensive intervention, integrating different components into a complex whole. Apart from the applies skills training, other components in the behavioral medicine physiotherapy intervention can be assumed to relate to the findings. The functional assessment of behavioral contingencies for the personal target activities was central in decision making for the treatment plans. In case of fear-related avoidance, tailored exposure components were integrated in the skills training. One potential benefit from the intervention might be decreased pain-related fear for some participants, as indicated in the quotations. Exposure therapy has been extensively evaluated in chronic pain and successfully implemented in physiotherapy treatment [30,74]. Fear avoidance, catastrophizing and pain beliefs are all important contributors for disability in chronic pain [26,27,29], and could mediate intervention effects. All of these factors were potentially addressed in the behavioral medicine physiotherapy intervention, by pain education, physical exercises and exposure contingencies. Assessment of these variables would be needed, to study a broader spectrum of mediators of effect on target activity performance. Additional behavior change techniques (BCT’s) in this study protocol were goal setting, self-monitoring, problem solving, graded tasks and activities, and feedback. All of these are well-known BCT’s, and studies indicate larger effect sizes of behavior change with the application of greater number of BCT’s [75].

The results of the goal achievement, increased frequency or duration of target activities and reported decrease in disability in the activity (the activity performance item) indicate that function in target activities improved during the intervention period. In the descriptive measures, a pattern of improvement in the Pain Disability Index can be observed for the two participants who rated high performance for the target activities. Pain intensity ratings were similar between baseline and post-intervention, except for participant P3, who had decreased pain intensity. Hence, results indicate that the intervention might affect task-specific disability, but to a lesser extent pain intensity. The written reports from the participants at the end of the exercise intervention were used as examples of enactment of skills [49]. A preliminary interpretation of the quotations is that the individuals might still suffer from the pain, but they were not restricted to the same extent. This would need to be further elaborated with different research methods to illuminate the processes of change.

The physiotherapy intervention included moderate to high-intensity strength training, which, based on the monitoring, was achieved for two of the participants (P1 and P3), but maybe not for the third (P5). In previous research, positive effects of aerobic and resistance training on physical function and disability have been demonstrated in chronic pain conditions, although there is no evidence of one program being superior to the others [36,76]. Beneficial outcomes for individuals with chronic pain can be achieved from protocols that allow for pain during exercise [55,76]. Physical exercise in the presence of chronic pain can be challenging, but pain need not be a barrier to exercise, which was supported in the current physiotherapy intervention. The general aerobic and strength training program in this study was not specifically related to the target activities but aimed to ensure an evidence-based level of physical exercise to improve physical function and health. All participants who completed the study achieved the physical exercise quota. However, the demands of the general exercise program might have contributed to dropouts, and other combinations of regular physical exercise and the components tailored to target activities should be explored.

The ratings of work ability did not change consistently in relation to the behavioral medicine physiotherapy intervention and sometimes displayed a large variability. One reason might be that work ability is affected by a variety of factors, for example, health, competence, and workplace factors [77]. For work ability, a pattern of improvement could be seen over the full study period, meaning it was not under experimental control from the design and no conclusions about effect could be drawn. The potential effects of the workplace components and of the combined intervention on work ability need to be further studied with other research designs. Beforehand, it was hypothesized that improvement in target activities could be of importance for the daily perception of work ability. This specific relationship was not supported by the results. Rather, one could consider further exploration of effect of combined interventions, such as this one, on work ability, and its relationship to RTW. Multi-component intervention with active workplace involvement for individuals with chronic pain on long term sick leave is supported by the literature [8,10,12,13,16,17], and formed the larger context for this study. Research into experienced challenges in RTW for individuals with chronic pain highlight the importance of both individual and workplace factors, supporting further exploration of combined interventions [78].

Behavioral medicine physiotherapy is based on a biopsychosocial understanding and treatment, which share key components with other approaches of combining physical exercises with psychosocial interventions, such as psychologically informed physiotherapy or, functional behavioral therapy [33,79,80]. Two recent reviews, on chronic and musculoskeletal pain and biopsychosocial interventions in primary care [33] and psychologically informed physical therapy [79], conclude that a larger incorporation of psychosocial factors could be favorable compared to exercise only, and the need for further research is stressed. A question arises about requirements of training, and barriers to a behavioral medicine treatment approach among physiotherapists. It is evident that physiotherapists’ beliefs and attitudes affect treatment, and that there are challenges in implementation [81,82,83]. Apart from training, support in terms of supervision and manuals seems crucial [33,83,84].

### 4.1. Methodological Considerations

A single-case experimental design provides the ability to assess within and between person variability and predicted mediators of change [44]. In this study, we have gained detailed insight into ratings of target activities and work ability over time for each participant. These can be hypothesized as process variables for pain-related disability at work and return-to-work outcomes.

Methodological strengths of this study include a structured analysis and replication across participants with varying lengths of baseline. Experimental control in SCED can be enhanced by replication across participants with varied introduction of the experimental phase, in concurrent or non-concurrent multiple baseline designs [45]. A minimum of three demonstrations of effect are warranted. In this study, there was a small variation in lengths of baseline, which limits the experimental control of the design. All three participant who completed the study displayed improvements in the self-efficacy ratings, which strengthens the interpretation of effect on this variable. Another limitation of this study is the lack of randomization procedures for the full study period, to provide opportunities for additional analytic techniques [45,85].

Visual analysis holds a long tradition in SCED research, and is considered of acceptable quality if conducted according to recommendations and aided by quantitative methods [85]. There are a number of challenges in the use of available quantitative methods in SCED, such as limitations in statistical inference [86]. In this study, the visual analysis formed the basis of analysis, and effect sizes from NAP and Tau-U were used to support interpretations. In the results of the nonoverlap measures, there was a pattern of larger effects in the NAP calculations than in the Tau-U, in cases with positive trends in phase A–B–C. This indicates that the Tau-U better controlled for baseline trend in data.

The fact that two of five participants dropped out from the behavioral medicine physiotherapy intervention introduces threats to internal validity and generalization. Dropouts indicates difficulties in engaging all eligible individuals in the behavioral medicine physiotherapy intervention, and that the demands of time, engagement and physical exercise was not feasible or attractive to all study participants. This raises questions on how the intervention can be modified to reach a larger proportion of individuals with chronic pain.

In SCED, there could be an effect of repeated measures on the outcome variables, for example, when data are collected by self-monitoring. Traditionally, this is controlled for by awaiting a stable baseline pattern before starting intervention phase [42], which was not possible in this study. Self-report was ongoing daily for two to three weeks of baseline, and nine to twelve weeks in total when the physiotherapy intervention was started, a time which could be considered enough for eventual effects of monitoring to stabilize.

Finally, this study, with an ABCD + follow-up design, has the potential for carry-over effects built in. The phases of workplace components formed the context for the behavioral medicine physiotherapy phase. Evaluation of this combined intervention was handled by observation of data patterns across and within all phases; A, B, C, D. The main research question was effect of the behavioral medicine physiotherapy as added to the workplace components; however, patterns and trends during the workplace phases were noted, and effect of the physiotherapy intervention should be interpreted in the context of the whole study protocol.

Training of intervention providers was standardized, and supervision provided regularly, which are suggested as essential for treatment integrity [49]. It could be stated that the intervention was delivered face-to-face for two of the participants (P1 and P3), whereas the third participant (P5) had a home-based exercise program and received coaching online and via phone calls. This type of delivery of the intervention also seemed feasible; however, it might have affected the structured exercise in terms of feedback on intensity and performance of resistance exercises.

### 4.2. Implications and Future Research

Interventions in the RTW arena are complex, as defined by a large number of interaction components and stakeholders [44]. Feasible interventions with active workplace involvement are needed for individuals with chronic pain on long-term sick leave [11]. In this systematic replication study, behavioral medicine physiotherapy was successfully adapted to the RTW context, and the protocol can undergo further evaluation. Effects from the combined intervention on RTW outcomes should be evaluated by large scale randomized controlled research designs. However, the number of dropouts in this study should be considered, and efforts made in future research to explore reasons and elaborate on feasibility and participation rates. Based on the literature on combining workplace involvement and physiotherapy and/or psychological treatment for RTW for individuals with chronic pain [8,11,12,13,14,16,17], we argue that there is a potential for integration of work-directed behavioral medicine physiotherapy in RTW rehabilitation, and further research is warranted. Physiotherapists are suitable to play an important role in work ability assessment and interventions for patients on long-term sick leave, and there has been a call for more research on work-directed physiotherapy interventions [87]. Clinical implications of this study are the illumination of the relationship between task-specific self-efficacy and increased function in target activities, where self-efficacy can be interpreted as a potential mediator of treatment effect. Another implication is the importance of the applied skills training component in behavioral medicine physiotherapy treatment.

## 5. Conclusions

Behavioral medicine physiotherapy for persons with chronic pain can be successfully adapted from previous protocols and replicated in an RTW context. Behavioral medicine physiotherapy seems to improve task-specific self-efficacy for target activities at work, which was supported by goal achievement and global improvement. The physiotherapy intervention, however, did not consistently affect the experience of target activities or work ability. Further large-scale studies where behavioral medicine physiotherapy is combined with active workplace involvement are warranted to evaluate the effects on RTW outcomes and pain-related disability. Personally prioritized activities at work, linked to work tasks, provide a meaningful basis for goal setting and skills acquisition for individuals with chronic pain and work disability, which could be of use in work-directed rehabilitation.

## Figures and Tables

**Table 1:** Overview of study design and measures.

Phase	A	B	C	D	Follow-Up
	Baseline	Return-to-Work (RTW) Coordination	Effective Communication within the Organization (ECO)	Behavioral Medicine Physiotherapy	Post-Intervention, 1 month and 2 months
**Description**		Workplace component with assessment by coordinator and workplace meeting	Workplace component with training and workplace meeting	Structured exercise with behavioral skills training, tailored to personalized target activities at work	
**Length in weeks**	2–3	2–5	3–5	14–18	2
**Repeated ratings of target activities and work ability**	X	X	X	X	X
**Goal achievement**	X				X
**Descriptive** **pre- and post- measures**	X				X
**Fidelity**		X	X	X	

**Table 2:** Participant characteristics.

Participant	Employment	Target Activities at Work	Pain Duration (Years)	Pain Localization	Pain Related Diagnoses ICD-10
**1**	Student	Handwriting or computer work	4	Head and neck, radiating in shoulder and arm left side	R52.2C Chronic pain
**2**	Administration	Handwriting or computer work Lifting	1.5	Hand and forearm right side	R52.2C Chronic pain, M13.0 Polyarthritis
**3**	Health care professional	Seated work Lifting	1.5	Pelvic	R52.2C Chronic pain, R10.2 Pelvic and perineal pain
**4**	Social work	Driving a car Lifting	>10	Neck, back, legs, hands on both sides	R52.2C Chronic pain, M79.7 Fibromyalgia
**5**	Administration	More demanding work tasks in the afternoon	2.5	Neck	R52.2A Chronic pain, M79.1 Myalgia

**Table 3:** Sick leave, return to work and work ability for all participants at baseline, post-intervention and at 2-month follow-up.

Participant	Measure	Baseline	Post- Intervention	2-Month Follow-Up	6-Month Follow-Up
1	Sick leave ^a^	50%	50%	50%	50%
	Studies	50%	66% ^b^	66% ^b^	66% ^b^
	Self-efficacy to increase work time 25% ^c^	2	3	3	
	WAI ^d^	18	28	33.5	
2	Sick leave	75%	75%	50%	0%
	Work	25%	25%	50%	100%
	Self-efficacy to increase work time 25% ^c^	1	1.5	1	
	WAI ^d^	24	30	16	
3	Sick leave	75%	0%	0%	0%
	Work	25%	75%	75%	75%
	Self-efficacy to increase work time 25% ^c^	3	1.5	2	
	WAI ^d^	23	29	34	
4	Sick leave	50%	0%	0%	50%
	Work	50%	70%	100%	50%
	Self-efficacy to increase work time 25% ^c^	2	2.5	NA	
	WAI ^d^	24	29	26	
5	Sick leave	25%	25%	25%	25%
	Work	75%	75%	75%	75%
	Self-efficacy to increase work time 25% ^c^	1	1	1	
	WAI ^d^	29	25	26.5	

^a^ Percentage of paid sick leave from the Swedish social insurance agency or correspondingly. ^b^ Participant 1 effectively studied 66% in terms of credits/semester. ^c^ Scores: 1 = very uncertain, 2 = somewhat uncertain, 3 = quite confident, 4 = completely confident. Not applicable when work = 100%. ^d^ Work Ability Index scores: range 7–49, 7–27 = poor, 28–36 = moderate, 37–43 = good, 44–49 = excellent work ability.

**Table 4:** Pain Disability Index scores, pain intensity, and physical activity for all participants at baseline, post-intervention and at 2-month follow-up.

Participant	Measure	Baseline	Post- Intervention	2-Month Follow-Up
1	Pain Disability Index ^a^	42	33	33
	Pain Intensity ^b^	6.5	7	6.5
	Average steps/day, M (SD) ^c^	8436 (1887)	7457 (1310)	8119 (1271)
2	Pain Disability Index ^a^	54	40	38
	Pain Intensity ^b^	4.5	7	7.5
	Average steps/day, M (SD) ^c^	10,646 (1080)	8637 (1589)	9358 (2193)
3	Pain Disability Index ^a^	34	19	19
	Pain Intensity ^b^	5	3	2.5
	Average steps/day, M (SD) ^c^	12,400 (2977)	10,673 (1352)	9963 (1237)
4	Pain Disability Index ^a^	50	43	37
	Pain Intensity ^b^	7.5	6	8
	Average steps/day, M (SD) ^c^	12,529 (2169)	12,843 (1744)	15,320 (1133)
5	Pain Disability Index ^a^	39	35	38
	Pain Intensity ^b^	8	9	8
	Average steps/day, M (SD) ^c^	11,921 (1501)	11,782 (1726)	14,407 (2527)

^a^ Pain Disability Index ranges from 0 to 70, with higher scores reflecting higher interference of pain with daily activities. ^b^ Daily ratings of pain worst pain intensity on numeric rating scale where 0 = no pain and 10 = pain as bad as you can imagine. Median score from a 2-week time period. ^c^ Assessed by ActiGraph GT3x accelerometer 24 h of the day for 10 days.

**Table 5:** Results from repeated ratings of target activities and work ability for participants 1, 3 and 5.

Participant and Measure	Phase A–B–C	Phase D	Follow-Up	Exercise Phase D vs. Phase A–B–C		Follow-Up vs. Phase A–B–C	
	Median (Range min-max)	Median (Range min-max)	Median (Range min-max)	NAP ^a^	Tau-U ^b^	NAP ^a^	Tau-U ^b^
P1 Handwriting or computer work, experience	4.5 (3–5)	5 (3–6.5)	5.5 (4–6)	0.73	0.45	0.76	0.51
P1 Handwriting or computer work, self- efficacy *	4 (1–6)	6 (4–8)	7 (6–8)	0.85	0.65	0.98	0.80
P1 Work ability *	4.25 (3.5–5)	4.5 (3.5–6)	6 (4.5–6)	0.56	−0.05	0.78	0.06
P3 Seated work, experience	5 (3–6.5)	5.5 (4–8)	7.5 (7–8)	0.65	0.30	1	1
P3 Seated work, self-efficacy	5 (5–8)	7 (4–8)	8 (8–9)	0.73	0.46	0.97	0.93
P3 Work ability	6 (2–8)	7 (4–8.5)	8.5 (7.5–9)	0.73	0.45	0.97	0.93
P3 Lifting, experience	4.5 (2–5)	6.25 (3–9)	8.5 (8–9)	0.88	0.75	1	1
P3 Lifting, self-efficacy	5 (5–7)	7 (2–9)	9 (9–9)	0.74	0.48	1	1
P5 Work tasks in the afternoon, experience *	3 (2.5–4)	4 (3–5)	4.5 (3.5–5)	0.86	0.60	0.96	0.48
P5 Work tasks in the afternoon, self-efficacy *	2 (1–4)	4 (1–5)	4.5 (4–5)	0.77	0.42	0.99	0.65
P5 Work ability *	5.75 (4–6)	7 (6–7)	6.75 (6–7)	0.96	0.65	0.94	−0.08

^a^ Nonoverlap of all pairs, values between −1 to 1 representing proportion of data separation between phases. ^b^ Tau-U, values between −1 to 1, representing proportion of data separation between phases. * Indicates correction for baseline trend has been applied.

**Table 6:** Goal achievement for target activities and self-rated improvement for participants 1, 3 and 5. Description of treatment content.

Participant	Target Activity	Measure	Baseline	Post- Intervention	2-Month Follow-Up
1		Clinical improvement ^a^		6	7
	Handwriting or computer work	Satisfaction with ability ^b^	1	7	8
		Activity performance ^c^	8	3	3
Goal	To write for 45 min	Duration ^d^	15	45	45
Extended goal	To write for 3 h			Achieved	
Functional behavioral analysis	Avoidance of target activity due to fear of pain, and limited coping strategies.				
Interventions	Physical exercise, exposure and graded activity - Exercises for physical function in neck, back and shoulders - Identification of thoughts and actions - Mini-breaks and other coping strategies - Application of strategies in target activity practiced in different situations - Sub-goals and gradually increased duration of target activity				
3		Clinical improvement ^a^		6	6
	Seated work	Satisfaction with ability ^b^	3	7	9
		Activity performance ^c^	6	2	2
Goal	To be able to do the work task, according to needs	Duration ^c^	60	155	147.5
Extended goal	To sit on a stool when performing clinical exams			Achieved	
Functional behavioral analysis	Avoidance of target activity due to fear of pain.				
Interventions	Graded exposure - Identification of thoughts and actions - Exposure for sitting practiced during training sessions and at home, with increased difficulty and duration - Exposure exercises in applied context at work				
	Lifting	Satisfaction with ability ^b^	3	9	8
		Activity performance ^c^	6	1	3
Goal	To be able to do the work task, according to needs	Frequency ^e^	0	1.5	6
Functional behavioral analysis	Avoidance of target activity due to fear of pain. Decreased physical capacity related to lifting.				
Interventions	Physical exercise, exposure and graded activity - Exercises activating pelvic muscles - Identification of thoughts and actions - Lifting exercises in the gym with increased load to improve physical function - Lifting in applied context at work, related to work tasks				
5		Clinical improvement ^a^		5	5
	Work tasks in the afternoon	Satisfaction with ability ^b^	0	4	5
		Activity performance ^c^	9	6	5
Goal	To be able to do more demanding work tasks in the afternoon	Proportion ^f^	30%	71%	83%
Functional behavioral analysis	Avoidance of target activity due to fear of pain. Avoidance of movements involving neck rotation and decreased range of motion.				
Interventions	Physical exercise, exposure and graded activity - Exercises for physical function in neck, back and shoulders - Identification of thoughts and actions - Practice of self-validation and self–regulation of difficulties arising - Sub-goals and gradually increased frequency of target activity				

^a^ Perception of change of health status. Measured with the Patient Global Impression of Change where 1 = very much worse, 2 = much worse, 3 = minimally worse, 4 = no change, 5 = minimally improved, 6 = much improved, 7 = very much improved. ^b^ Self-rated satisfaction with ability for the target activities, NRS 0–10 where 10 = “very satisfied”. Measured with The Patient Goal Priority Questionnaire (PGPQ). ^c^ Self-rated performance of the target activity, NRS 0–10 where 0 = “no disability”, measured with the PGPQ. ^d^ Duration in minutes. Median of 2 weeks daily measures. ^e^ Frequency in numbers of lifts at work per day. Median of 2 weeks daily measures. ^f^ Proportion of days performing more demanding work tasks in the afternoon during a 2-week period. Data from daily measures.

**Table 7:** Delivery of intervention: Adherence to physical exercise in the behavioral medicine physiotherapy intervention for participant 1, 3 and 5.

Participant	Adherence to Number of Exercise Sessions % (n/total)	Adherence Sets/Reps per Exercised Session %	Self-Reported Exercise Challenge ^a^ Md (min–max)	Adherence Intensity Resistance Exercises ^b^ %	Adherence Intensity Aerobic Training ^c^ %
1	93% (28/30)	96%	8.5 (7–10)	100%	100%
3	90% (27/30)	100%	8 (5–9)	78%	100%
5	85% (22/26)	100%	6 (4–8)	46%	missing

^a^ NRS-rating 0–10 on the Omni-scale of Resistance Exercise, from “extremely easy” to “extremely challenging”. ^b^ Rating of 7 or above on the Omni-scale of Resistance Exercise NRS 0–10. ^c^ Rating of 11 or above on the Borg RPE scale NRS 6–20.

## Data Availability

The data presented in this study are available upon reasonable request from the corresponding author.

## Supplementary Materials

The following are available online at https://www.mdpi.com/article/10.3390/ijerph19031509/s1, Table S1. Content of the Behavioral Medicine Physiotherapy Intervention. Structured according to The TIDieR (Template for Intervention Description and Replication) Checklist.
