# Supplementary material for: Behavioral Medicine Physiotherapy in the Context of Return to Work for Chronic Pain: A Single-Case Experimental Design Study
Source: Int J Environ Res Public Health. 2022 Jan 28;19(3):1509. doi: 10.3390/ijerph19031509 (PMC8835028; doi:10.3390/ijerph19031509)
Supplement: Supplementary file 1 [file ijerph-19-01509-s001.zip › ijerph-1492994-supplementary.pdf]

Supplementary Materials

Table S1. Content of the Behavioral Medicine Physiotherapy Intervention. Structured according to The TIDieR (Template for Intervention Description and Replication) Checklist.

NAME	Behavioral Medicine Physiotherapy
WHY	**Aim:** To decrease disability and improve target activity performance.<br>In this study, specifically, to improve pain-related disability at work and target activities linked to work tasks.<br><br>**Theory:** Behavioral medicine is theoretically based on the interaction between behavioral and biomedical factors, relevant to health and disease. In behavioral medicine physiotherapy these principles guide assessment and treatment, meaning physical, psychosocial and behavioral components all are in focus.<br><br>In the treatment approach for individuals with disabilities in activities in everyday life, a core feature is to identify individually prioritized target activities, to which assessment and treatment is tailored.<br><br>**Essential elements:** The treatment is hypothesized to increase function in activities in everyday life by a combination of improved prerequisites, such as physical function, and improved skills in and levels of the target activities. To reach this goal, behavior change strategies are combined with physical exercise. Tailored behavioral skills training is a key component. An additional component was to provide an evidence-based level of physical exercise.
WHAT	**Procedures:** Personalized target activities at work formed the basis for the intervention. The program consisted of general aerobic and strength training, and tailored basic and applied skills training based on goal-setting and analysis of the target activities. Training was supervised by a physiotherapist. Strategies for long-term maintenance were incorporated. Detailed description of the steps can be found under Tailoring.<br><br>**Materials:** Participants received diary for self-monitoring of physical exercise and target activities and its contingencies. They received home-assignments and follow-up on sub-goals. Participants also received a tool for exercise monitoring, a Huawei accelerometer. Personnel received treatment manuals based on the steps described in "Tailoring" and checklists for delivery.
WHO PROVIDED	Physiotherapists with several years of clinical experience and special training in in behavioral medicine physiotherapy provided the intervention. Training included a post-graduate university course (credits corresponding to five weeks of full-time studies). Supervision was provided regularly within the research team.
HOW	Participants performed individual exercise programs in group sessions with supervision from a physiotherapist. In addition, individual follow-up was provided. The intervention was provided face-to-face. However, due to covid-19 outbreak, online meetings and home-exercises were provided for one participant.

WHERE	The intervention was provided at a training facility.
WHEN AND HOW MUCH	Participants took part of a 14-week period of structured exercise and tailored skills training.<br><br>**General aerobic and strength training:** Participants were attending two exercise sessions a week, about 75 minutes each. The general aerobic exercise consisted of 20 min of moderate intensity (approximate 70 % of max heart rate). The general strength training consisted of a whole-body approach including leg, upper body pull and push, shoulder and core exercises. Recommended intensity of strength training was as follows: 15 repetition maximum (RM) week 1-4, 8-12 RM week 5-8 and 6-8 RM week 9-14.<br><br>**Tailored exercises and behavioral skills training:** Every second week a 20-30 minutes individual follow-up sessions were held, where individual exercises and target activities, and home assignments and goal completion were discussed.
TAILORING	The analysis and tailoring were structured in the following steps:<br><br>**Identification of personalized target activities at work:** These were activities at work, necessary for work tasks, in which the participant perceived hindered due to disabilities. For example, sitting, lifting, writing. The following assessment and treatment were tailored to these activities.<br><br>**Assessment and analysis:** Participants monitored the target activities and their contingencies (i.e. context, behavior, and associated thoughts and feelings before and after), as a basis for functional behavioral analysis. Physiotherapists integrated the behavioral and psychosocial findings with analysis of physical function in target activities, in a behavioral medicine approach.<br><br>**Goal setting of target activities:** Goal-setting of target activities was specified and agreed upon between the participant and the physiotherapist, and new goals could be added.<br><br>**Basic skills training:** Based on the analysis of physical function and functional behavioral analysis for target activities, exercises for basic skills training were chosen by the physiotherapist and the participant together. Basic skills training includes acquisition of skills required in the target activity, practiced separately outside of everyday context. Both physical and psychological components were targeted, i.e. the physical aspect could be strength and the psychological aspects exposure for pain-related fear and identification of thoughts and strategies. Examples included neck- and shoulder exercises, lifting weights, sitting, taking breaks, etc. Individual exercises were practiced during the scheduled exercise sessions or as home assignments.<br><br>**Applied skills training:** Applied skills training includes real-life practice of the basic skills in the everyday life target activities, here, in the target activities

	at the workplace. That is, components of the target activity, previously practiced separately, are now practiced in the actual activity. Examples included exposure of pain-related fear, application of self-regulation strategies, and physical activities such as lifting or stretching.<br>In addition, applied skills training includes graded increase of the target activities per se, such as amount or level of difficulty of the activity. Examples included sub-goals of increased time of the target activity “writing”, and number of performances of the target activity “work tasks during PM”, and increased difficulty of the target activity “lifts at work”.<br><br>**Generalized skills training:** This step is similar to the applied skills training, and includes generalization over different contexts and activities.<br><br>**Maintenance and relapse prevention:** At the end of the intervention, the participants reflected in a written plan on strategies for maintenance of skills and target activities, including a plan for relapse prevention. Also, at the end of the intervention period, participants were encouraged to independently develop target activity performance and new sub-goals, as a strategy to enhance self-regulation and maintenance.<br><br>**Regular evaluation and feedback:** The participant and the physiotherapist evaluated the training, home assignments and goals regularly, and feedback was provided.<br><br>In the general aerobic and strength training program described above, exercises were adjusted to the individual. The participants and physiotherapists evaluated the intensity at a weekly basis. In addition, behavior change strategies were directed towards the implementation of a habit of regular physical exercise; self-monitoring of contingencies for physical exercise, and plan for maintenance and relapse prevention.
MODIFICATIONS	During the study period, the following changes were made to the intervention protocol, based on intermediate analyses of target activities and goal-setting: Extended intervention period for participants 1 and 3, more structured sub-goals of graded increase of target activities, modified home assignments for target activities, and weekly feedback to participants with their own data on target activity frequency/duration and experience. In addition, modification to online delivery for one participant was done due to the Covid-19 pandemic.
HOW WELL	Fidelity to the intervention protocol, and adherence among participants, were assessed by study logs, exercise programs and a diary for home assignments. Delivery of the intervention was consistent with the protocol. Three of five participants fulfilled the study protocol, and among those, adherence to the scheduled exercise sessions were good. Details can be found in Table 7.
